# PX-478, an HIF-1α inhibitor, impairs mesoCAR T cell antitumor function in cervical cancer

**DOI:** 10.3389/fonc.2024.1357801

**Published:** 2024-02-15

**Authors:** Ahmad Reza Panahi Meymandi, Behnia Akbari, Tahereh Soltantoyeh, Zahra Shahosseini, Mina Hosseini, Jamshid Hadjati, Hamid Reza Mirzaei

**Affiliations:** ^1^ Department of Medical Immunology, School of Medicine, Tehran University of Medical Sciences, Tehran, Iran; ^2^ Department of Medical Biotechnology, School of Allied Medical Sciences, Iran University of Medical Sciences, Tehran, Iran; ^3^ Virology Department, Pasteur Institute of Iran, Tehran, Iran; ^4^ Department of Pharmaceutical Biotechnology, School of Pharmacy, Tehran University of Medical Sciences, Tehran, Iran

**Keywords:** CAR T cell therapy, pharmacological targeting, HIF-1α, PX-478, cervical cancer, T cell exhaustion

## Abstract

**Introduction:**

Chimeric Antigen Receptor (CAR) T cell therapy has demonstrated remarkable success in treating hematological malignancies. However, its efficacy against solid tumors, including cervical cancer, remains a challenge. Hypoxia, a common feature of the tumor microenvironment, profoundly impacts CAR T cell function, emphasizing the need to explore strategies targeting hypoxia-inducible factor-1α (HIF-1α).

**Methods:**

In this study, we evaluated the effects of the HIF-1α inhibitor PX-478 on mesoCAR T cell function through in-silico and *in vitro* experiments. We conducted comprehensive analyses of HIF-1α expression in cervical cancer patients and examined the impact of PX-478 on T cell proliferation, cytokine production, cytotoxicity, and exhaustion markers.

**Results:**

Our in-silico analyses revealed high expression of HIF-1α in cervical cancer patients, correlating with poor prognosis. PX-478 effectively reduced HIF-1α levels in T and HeLa cells. While PX-478 exhibited dose-dependent inhibition of antigen-nonspecific T and mesoCAR T cell proliferation, it had minimal impact on antigen-specific mesoCAR T cell proliferation. Notably, PX-478 significantly impaired the cytotoxic function of mesoCAR T cells and induced terminally exhausted T cells.

**Discussion:**

Our results underscore the significant potential and physiological relevance of the HIF-1α pathway in determining the fate and function of both T and CAR T cells. However, we recognize the imperative for further molecular investigations aimed at unraveling the intricate downstream targets associated with HIF-1α and its influence on antitumor immunity, particularly within the context of hypoxic tumors. These insights serve as a foundation for the careful development of combination therapies tailored to counter immunosuppressive pathways within hypoxic environments and fine-tune CAR T cell performance in the intricate tumor microenvironment.

## 1 Introduction

Cervical cancer (CC) ranks as the fourth most prevalent cancer among women (1). Despite the existence of various preventive and treatment modalities for CC, such as HPV screening, prophylactic vaccines, surgical interventions, radiotherapy, and chemotherapy, the global burden of the disease remains substantial (2). Therefore, there is an urgent need for new treatment strategies to improve the prognosis of patients with CC.

In recent years, the development of chimeric antigen receptor (CAR) T cell therapies for treating solid tumors has garnered significant interest (3). Currently, numerous clinical trials are underway to assess the effectiveness of CAR-T cell therapy in cervical cancer patients (NCT01583686) (NCT04556669) (NCT03356795). Mesothelin (MSLN) stands out as a crucial target antigen in the pursuit of novel immunotherapies for solid tumors (4). Clinical trials involving anti-MSLN CAR T cells have demonstrated commendable safety profiles but limited efficacy (5). The tumor microenvironment (TME) is widely recognized as a major obstacle, as it impedes T cell survival, proliferation, and cytotoxicity, thereby limiting the application of CAR T cell therapies in the clinical management of solid tumors (6). A common feature of the TME is hypoxia, characterized by inadequate oxygen supply (less than 2% O2) due to heightened metabolic demands and inefficient vasculature, in stark contrast to healthy tissues with oxygen levels of 5%–10% (7). Hypoxia is clinically associated with a poor prognosis and resistance to chemotherapy and radiotherapy (8–10) and it significantly compromises the fitness and efficacy of CAR T cells (11).

At the heart of the cellular response to hypoxia is hypoxia-inducible factor-1 (HIF-1), a master transcription factor that orchestrates the regulation of numerous downstream targets (12). HIF-1 exists as a heterodimeric transcription factor composed of the oxygen-sensitive HIF-1α subunit and the constitutively expressed HIF-1β (ARNT) subunit (13). Under normal oxygen conditions (normoxia), HIF-1α is rapidly degraded through ubiquitin-mediated pathways, primarily governed by proline hydroxylation (14). However, during hypoxia, the inhibition of HIF-1α hydroxylases interferes with VHL-HIF binding, leading to the stabilization of HIF-1α protein and enabling HIF-1 dimerization, which, in turn, activates its transcriptional function (14).

Numerous studies have unveiled the connection between HIF-1α overexpression and poorer prognosis in cervical cancer patients (15–18). Currently, several HIF-1α inhibitors are in development for various cancer types, exhibiting promising antitumor efficacy and manageable toxicity profiles (19–21). Nonetheless, it remains uncertain whether agents that inhibit HIF-1α can enhance the response to CAR-T cell therapy. To elucidate these questions, we explore the impact of the selective HIF-1α inhibitor PX-478 on the antitumoral function of second-generation mesoCAR T cells.

## 2 Material and methods

### 2.1 Bioinformatics analysis

Gene expression analysis was conducted using the GEPIA2 database (http://gepia2.cancer-pku.cn), which utilizes data from the TCGA and GTEx databases (22). To compare HIF-1α gene expression levels between squamous cell carcinoma of the cervix (SESC) and corresponding normal tissues, the “box plot” function for expression analysis was employed. The following statistical parameters were utilized: a Log2FC (Logarithm to the base 2-fold change) cutoff value of 1, and a p-value cutoff value of 0.01. Additionally, GEPIA2 was employed to assess the overall survival of SESC patients using the “Survival Analysis” module, with the Group Cutoff set to the Quartile. Hazard ratios (HRs) with 95% confidence intervals (CIs) and log-rank P-values were computed to ascertain survival outcomes. The representative immunohistochemistry image of HIF-1α expression was obtained from the Human Protein Atlas (HPA) database (23).

### 2.2 Cell lines

HEK293T, Jurkat, Hela, and PANC-1 cell lines were acquired from the Iranian Biological Resource Center (IBRC). HEK293T, Hela, and PANC-1 cells were maintained in D10 media, comprising DMEM (Gibco, Life Technologies), 10% fetal bovine serum (FBS), and 1% penicillin/streptomycin (Gibco, Life Technologies). Jurkat cells were cultured in R10 media, containing RPMI-1640 (Gibco, Life Technologies) supplemented with 10% FBS, 25 mM HEPES (Sigma Aldrich), 2 mM glutamine (Gibco), and 1% penicillin/streptomycin. Flow cytometry was used to validate mesothelin expression in the relevant cell lines prior to experiments. Regular mycoplasma contamination checks were conducted on all cell lines.

### 2.3 Primary human cells

Peripheral blood mononuclear cells (PBMCs) were isolated from fresh blood using standard methods with Histopaque^®^-1077 (Sigma Aldrich). Primary human T cells were negatively selected with immunomagnetic beads (Pan T Cell Isolation Kit, Miltenyi Biotec) and stored at -80°C. T cells were cultured in TM10 media, composed of TexMACS™ Medium (Miltenyi Biotec), supplemented with 10% human serum and 100 IU/mL premium-grade rhIL-2 (Miltenyi Biotec). Blood samples were obtained from healthy volunteers under approval from the Research Ethics Committees of the School of Medicine, Tehran University of Medical Sciences [IR.TUMS.BLC.1402.015].

### 2.4 Lentiviral vector production

Lentiviral vectors were produced following previously established protocols (24). HEK293T cells were transfected with lentiviral CAR and packaging plasmids using the calcium phosphate method. Lentiviral supernatants were collected at 48- and 72-hour time points post-transfection and then concentrated through high-speed centrifugation. The concentrated lentivirus batches were resuspended in cold RPMI-1640 media and stored at -80°C. Titration of lentiviral vectors was performed using Jurkat cells.

### 2.5 Lentiviral transduction

mesoCAR T cells were generated as per previous descriptions (25). Briefly, 1 × 10^6^ T cells were seeded in each well of 12-well tissue culture plates and activated using Dynabeads™ Human T-Expander CD3/CD28 (Gibco, Life Technologies, 11161D) at a 1:1 ratio in TM10 media. Activated T cells were infected with lentiviral vectors supplemented with 8 mg/mL Polybrene (Santacruz) 24 hours after activation. Centrifugation at 850g for 1 hour at 32°C was employed to enhance transduction efficiency. Two hours later, 2 mL/well of TM10 media was added to the transduced T cells. At day 4 post-transduction, Dynabeads™ were removed from transduced T cells using a DynaMag™ magnet, and GFP expression, indicative of mesoCAR expression, was assessed via flow cytometry.

### 2.6 PX-478 dose-response

PX-478 (MedChemExpress, USA) was dissolved in Dimethyl sulfoxide (DMSO). To assess the impact of PX-478 on T cell proliferation, 2 × 10^5^ CFSE-labeled T cells were seeded in 96-well tissue culture plates and exposed to varying concentrations of PX-478. Human T Cell-Expander Dynabeads™ CD3/CD28 (Gibco, Life Technologies, 11161D) were used at a 1:1 ratio in TM10 medium to activate T cells. After three days, T cells were harvested, and their proliferation was evaluated via flow cytometry.

### 2.7 Protein extraction and western blotting

Adherent cells were washed twice with PBS, scraped, and transferred to 1.5 ml tubes. T cells were also harvested and washed twice with PBS. After centrifugation, cells were lysed using RIPA buffer containing 1mM PMSF at a ratio of 60 μl per 10^6^ cells. Proteins were separated by 10% SDS-PAGE under reducing conditions and subsequently transferred to PVDF membrane. The membrane was then blocked for 1 hour using a 5% BSA blocking reagent in Tris-Buffered Saline (pH=7.5) containing 0.05% Tween-20 (v/v) (TBST) and incubated with Rabbit anti-HIF-1α antibody diluted at 1:2,000 (Novus Biologicals NB100-449, Centennial, Colorado, USA) or Rabbit anti-β-actin antibody (Sigma-Aldrich) diluted at 1:2,000, overnight at 4˚C. The blots were further incubated with anti-Rabbit horseradish peroxidase-conjugated antibodies for 1 hour. Protein bands were detected using ECL method and X-ray film was used for visualization. Quantification was conducted using image J (imagej.org).

### 2.8 Hypoxia assay

Culture plates were incubated either under normoxic conditions (37°C in humidified air, 5% CO2) or under hypoxic conditions (1% O2, 5% CO2, 94% N2). Hypoxia was induced using a hypoxia incubator chamber (StemCell Technologies, Inc.) purged at 25L/min for 4 minutes with a gas mixture containing 1% O2, 5% CO2, and 94% nitrogen as a balance before sealing the chamber.

### 2.9 *In vitro* cytotoxicity assay

For *in vitro* cytotoxicity assays, 1x10^4^ target cells were seeded in 96-well U‐bottomed tissue culture plates and pretreated with 25µM PX-478 for 24 hours under both hypoxic and normoxic conditions. Transduced or non-transduced T cells were then added to the wells at effector-to-target ratios of 1:1, 10:1, and 20:1 for 4 hours in TM10 media, with a final volume of 200 ml/well. To distinguish between effector and target cells, effector cells were stained with CFSE. Prior to flow cytometry analysis, 7-AAD (Miltenyi Biotec) was added to stain dead cells. Flow cytometry analysis utilized CFSE and 7-AAD staining to differentiate T cells from dead tumor cells. The frequency of lysed target cells (CFSE-/7-AAD+ cells) was calculated by subtracting the percentage of spontaneous lysis of target cells from the percentage of lysis of target cells in coculture with mesoCAR T cells. Normalized lysis of target cells (Specific lysis) was reported based on mesothelin expression on target cells.

### 2.10 *In vitro* proliferation and cytokine production assays

Target cells were treated with 50 mg/ml of mitomycin C (Sigma Aldrich) for 30 minutes at 37°C and subsequently washed. 2x10^5^ target cells were seeded in 48-well tissue culture plates and pretreated with 25µM PX-478 for 24 hours under both hypoxic and normoxic conditions before removal of the media. For cell proliferation analysis, mesoCAR T cells and untransduced T cells were stained with 5 mM CFSE at room temperature for 8 minutes. An equal amount of FBS was added to halt the reaction. After washing three times with complete RPMI 1640 medium, CFSE-labeled cells (0.2 × 10^6^/well) were cocultured with either target cells or media, in the absence of exogenous IL‐2, in 48‐well plates, with a final volume of 800 µl/well. After 24 hours, 200 µl of the supernatants were harvested and stored at −80°C. The subsequent cytokine analysis was carried out by enzyme-linked immunosorbent assay (ELISA) to quantify IFN-γ and IL-2. After 72 hours, cells were stained with PerCP-conjugated anti-human CD3 antibody (Clone: HIT3a, BioLegend), and CFSE dilution of CD3+ cells was determined by flow cytometry, as an indicator of proliferation.

### 2.11 Flow cytometric analysis

The purity of isolated T cells was confirmed using APC-conjugated anti-human CD3 (Clone: UCHT1, BioLegend). PE-conjugated anti-human mesothelin (Clone: #420411, R&D Systems) was used to detect mesothelin expression. FITC-conjugated anti-human CD3 (Clone: HIT3a, BioLegend), PE-conjugated anti-human CD279 (PD-1) (Clone: EH12.2H7, BioLegend), and APC-conjugated anti-human CD366 (Tim-3) (Clone: F38-2E2, BioLegend) antibodies were used to measure the expression of exhaustion markers. For proliferation assays, cells were loaded with CellTrace™ CFSE (Life Technologies, #C34554) according to manufacturer’s instructions, and T cells were detected using PerCP-conjugated anti-human CD3 antibody (Clone: HIT3a, BioLegend). Data were collected using a BD FACSCalibur (BD Biosciences) and analyzed with FlowJo software (v10.6). All assays were performed in duplicate and repeated two to three times.

### 2.12 Statistical analysis

Normality tests and one-way/two-way analysis of variance (ANOVA) were performed using GraphPad Prism software (v9) to identify differences among various treatment groups. p-values below 0.05 were considered statistically significant.

## 3 Results

### 3.1 Association of HIF-1α overexpression with adverse prognosis in cervical cancer patients

**Figure 1:**
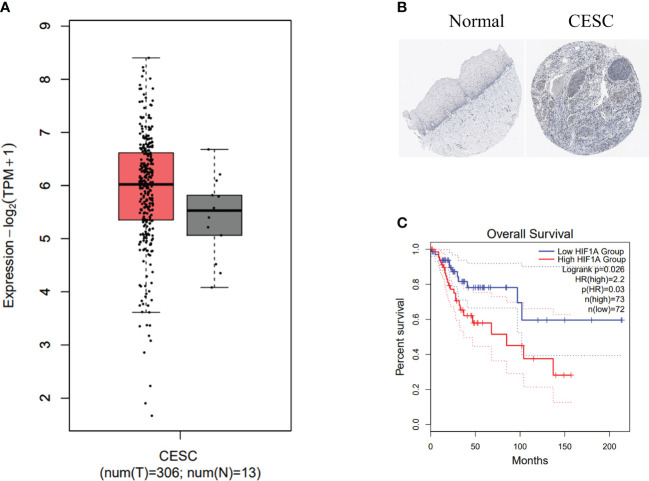
HIF-1α overexpression is associated with poor prognosis in cervical cancer patients. **(A)** HIF1A expression levels in biopsies from cervical cancer (CC) patients (highlighted in red) and corresponding normal tissue samples (depicted in grey) were analyzed using data from the TCGA dataset. The data was log2 transformed (TPM+1). **(B)** Representative images from the Human Protein Atlas database illustrate HIF-1α expression in normal (left) and CC (right) tissue samples. **(C)** Kaplan-Meier survival curves present the overall survival time of CC patients categorized into high and low HIF-1α expression groups. Dotted lines indicate a 95% confidence interval.

To assess the significance of HIF-1α expression in cervical cancer, we utilized the GEPIA2 database to visualize the mRNA expression levels of HIF-1α in cervical cancer. The analysis involved 13 normal tissue samples and 306 samples from cervical cancer patients. Our data unequivocally demonstrate an upregulation of HIF-1α in cervical cancer patients (**Figure 1A**). Complementing this, the immunohistochemistry image of HIF-1α protein levels in tissue samples from the Human Protein Atlas (HPA) dataset confirmed similar findings (**Figure 1B**).

We further conducted a Kaplan-Meier survival analysis using GEPIA2 to investigate the prognostic value of HIF-1α. Our results reveal a statistically significant association between high expression of the *HIF1A* gene and shorter overall survival in cervical cancer patients (**Figure 1C**).

### 3.2 PX-478 reduces HIF-1α protein levels under hypoxic conditions

**Figure 2:**
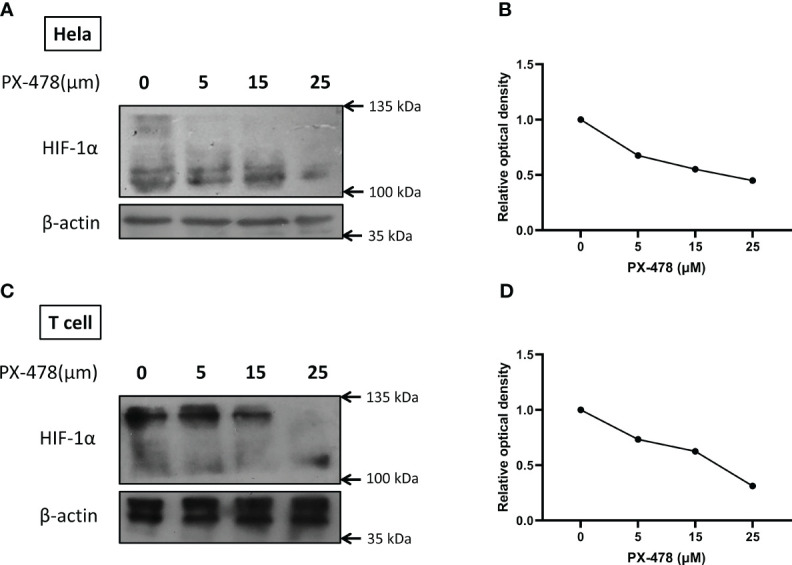
PX-478 decreases HIF-1α protein levels in a dose-dependent manner. **(A, C)** Hela and T cells were exposed to varying concentrations of PX-478 for 24 hours under hypoxic conditions, and the levels of HIF-1α protein were assessed through Western blot analysis. **(B, D)** PX-478 demonstrates a dose-dependent inhibition of HIF-1α protein expression. Densitometric quantification of the blots was performed relative to β-actin as a reference protein. Each experiment was repeated two to three times.

PX-478, previously identified as a compound that decreases cellular HIF-1α levels, was evaluated in our study. To validate the increase of HIF-1α under hypoxic conditions and assess the inhibitory effect of PX-478, HeLa and T cells were cultured under normoxic and hypoxic (1% O2) conditions while exposed to varying doses of PX-478 for 24 hours. Western blot analysis confirmed the efficient stabilization of HIF1α in hypoxic conditions and demonstrated that PX-478 inhibits the hypoxia-induced increase in HIF-1α protein levels in a dose-dependent manner (**Figures 2A-D**).

### 3.3 Effective antitumor activity of mesoCAR T cells against cervical cancer cells

**Figure 3:**
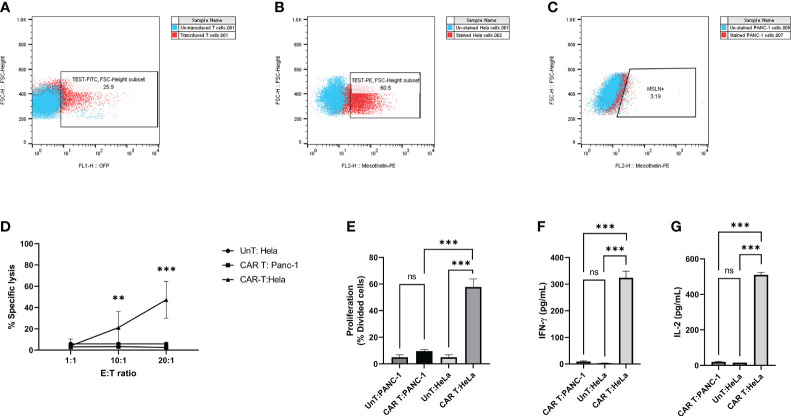
Antigen specificity of mesoCAR T cells against HeLa cells. **(A)** Assessment of chimeric antigen receptor (CAR) expression on mesoCAR T cells. **(B, C)** Representative dot plots illustrating mesothelin expression on HeLa and PANC-1 cells, respectively. **(D)** MesoCAR T cells demonstrate specific cytotoxicity against target cells at varying effector-to-target ratios. **(E)** Proliferation of mesoCAR T cells in response to target cells. **(F, G)** Production of IFN-γ and IL-2 by mesoCAR T cells in coculture with target cells. Statistical analysis was conducted using ordinary one-way ANOVA **(E–G)** and two-way ANOVA **(D)**, followed by Tukey’s multiple comparison test. Significance denoted by ***(P < 0.001). Data are presented as mean ± SD. ns, non significant

We generated and characterized second-generation mesoCAR T cells, as described previously (25). Briefly, human CD3+ T cells were efficiently infected with lentiviral particles encoding the second-generation mesoCAR transgene (**Figure 3A**). We subsequently assessed the *in vitro* antitumor capacity of these cells. We used PANC-1 and HeLa cells, which represent mesothelin-negative and positive tumor cells respectively (**Figures 3B, C**). T cells expressing the mesoCAR transgene exhibited specific cytotoxicity against mesothelin-positive HeLa cells, while no cytotoxicity was observed against mesothelin-negative PANC-1 cells (**Figure 3D**). To test the effectiveness of mesoCAR T cells against HeLa cells, we investigated their proliferation and their capacity to produce IL-2 and IFN-γ cytokines *in vitro*. mesoCAR T cells demonstrated a high mesothelin-specific proliferation rate comparable to untransduced T cells after being stimulated with Hela and PANC-1 cells (**Figure 3E**). After CAR T cell stimulation with HeLa, mesoCAR T cells showed high mesothelin-specific proliferation rates and produced large amounts of IFN-γ and IL-2, comparable to untransduced T cells (**Figures 3E-G**). No IL-2 and IFN-γ secretion was detected in cultures of T cells alone, tumor cells alone, or when irrelevant target cells like PANC-1 were involved (**Figures 3F, G**).

### 3.4 Impact of PX-478 on mesoCAR T cell proliferation

**Figure 4:**
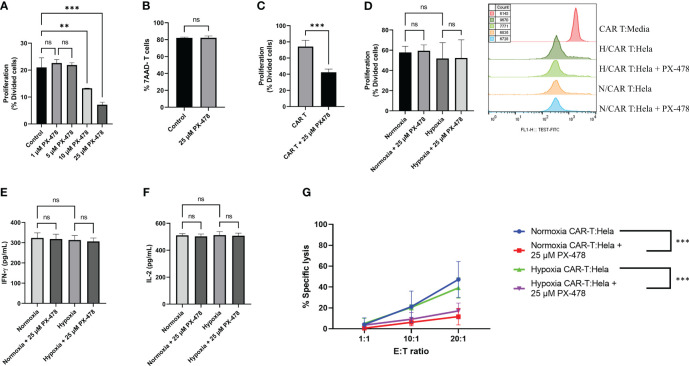
The effects of HIF-1α inhibitor PX-478 on the antitumor function of mesoCAR T cells. **(A)** PX-478 significantly inhibits antigen-nonspecific proliferation of T cells. **(B)** Viability of T cells remained unchanged in response to PX-478. **(C)** PX-478 significantly inhibits the IL-2-induced antigen-nonspecific proliferation of mesoCAR T cells. **(D)** Antigen-specific proliferative capacity and representative cell count of mesoCAR T cells over a three-day coculture with pre-treated Hela cells. **(E, F)** Production of IFN-γ and IL-2 by mesoCAR T cells in coculture with pre-treated Hela cells. **(G)** Overlaid plot demonstrating mesoCAR T cell cytotoxicity against Hela cells in the presence of PX-478. Statistical analysis was performed using ordinary one-way ANOVA **(A, C-F)**, Student’s t-test **(B)**, two-way ANOVA **(G)**, and Tukey multiple comparison test. **P < 0.01; ***P < 0.001. Data are presented as mean ± SD. ns, non significant

The efficacy of CAR T cell immunotherapies against solid tumors hinges on T cell proliferation, persistence, and accumulation (26). To investigate the influence of PX-478 on mesoCAR T cell proliferation, we performed a series of experiments. Initially, T cells activated with anti-CD3/CD28-coated beads were exposed to varying concentrations of orally available PX-478, revealing that PX-478 can dose-dependently reduce antigen-nonspecific T cell proliferation (**Figure 4A**), while concurrently maintaining T cell viability unchanged (**Figure 4B**).

Consistent with the data obtained from T cells, we observed that the antigen-nonspecific proliferation of mesoCAR T cells in an IL-2-containing medium was also impeded by PX-478 (**Figure 4C**). Given the observed direct inhibitory effect of PX-478 on T and mesoCAR T cell proliferation, we conducted experiments in which tumor target cells were pretreated with PX-478 for 24 hours under both hypoxia and normoxia conditions. After supernatant removal, mesoCAR T cells were introduced. Interestingly, pretreatment of tumor cells with PX-478 did not significantly impact mesoCAR cell proliferation (**Figure 4D**). This result was supported by the analysis of cytokine production, where no significant changes in the levels of IL-2 and IFN-γ were observed (**Figures 4E, F**). Collectively, our findings indicate that PX-478 directly inhibits antigen-nonspecific T and mesoCAR T cell proliferation, while pretreatment of tumor cells with PX-478 has no influence on mesoCAR T cell proliferation and cytokine production.

### 3.5 PX-478 impairs mesoCAR T cell cytotoxic function

Having established the effect of PX-478 on mesoCAR T cell proliferation, we sought to examine its impact on the cytotoxicity of these cells against tumor target cells. HeLa cervical cancer cells were cultured, pre-exposed to 25µM PX-478 for 24 hours, and then co-incubated with mesoCAR T cells under normoxic and hypoxic (1% O2) conditions. mesoCAR T cells demonstrated effective killing of HeLa cells under both hypoxic and normoxic conditions (**Figure 4G**). However, PX-478 significantly reduced the cytotoxicity of mesoCAR T cells under both hypoxic and normoxic conditions (**Figure 4G**).

In an effort to understand the underlying reason for the impairment of mesoCAR T cell cytotoxic function, we considered the possibility that these cells become exhausted in the presence of PX-478. Exhausted T cells can be categorized into progenitor-exhausted T cells (T_pex_) and terminally exhausted T cells (T_tex_) based on their function and phenotype (24, 27, 28). T_pex_ cells express PD-1 but not TIM3, retain stem-like characteristics, and remain polyfunctional. In contrast, T_tex_ cells express both PD-1 and TIM3 at high levels, have a limited lifespan, and cannot effectively suppress tumor growth (29). Immune checkpoint blockade can rejuvenate T_pex_ but not T_tex_ cells (30). To explore this, cells from our cocultures were analyzed via flow cytometry for the expression of PD-1 and TIM3. Our results revealed that pretreatment of tumor cells with PX-478 did not significantly alter the expression of PD-1 but led to an increased expression of TIM3 (**Supplementary Figure S1A, B**). Furthermore, the abundance of T_tex_ cells (PD-1+TIM3+) increased, while the abundance of T_pex_ cells (PD-1+TIM3-) decreased under both normoxic and hypoxic conditions (**Supplementary Figure S1C**).

## 4 Discussion

In this study, we delved into the impact of the HIF-1α inhibitor PX-478 on the antitumoral function of mesoCAR T cells. Our in-silico analysis compellingly indicated that the overexpression of HIF-1α in CC patients is strongly associated with an unfavorable prognosis. It is well-established that HIF-1α becomes stabilized within the hypoxic core of rapidly growing, poorly vascularized solid tumors (31). This stabilization of HIFs plays a pivotal role in promoting tumor survival and metastasis by orchestrating changes in glycolysis, nutrient uptake, waste disposal, angiogenesis, apoptosis, and cell migration (32–35).

To target HIF-1α, we employed PX-478, an orally available small molecule known to interfere with HIF-1α transcription and translation, thus leading to reduced deubiquitination of HIF-1α (36). Our Western blot analyses provided clear evidence that PX-478 effectively inhibited HIF-1α in a dose-dependent manner in both T and HeLa cells. Our investigation into the effects of PX-478 on T cell proliferation revealed a dose-dependent suppression. Notably, previous studies have shown that T cell receptor activation stabilizes HIF-1α in T lymphocytes (37), thereby facilitating a metabolic shift towards glycolysis to support T cell proliferation and effector functions (38, 39). Furthermore, PI3K/mTOR activity downstream of TCR and CD28 signaling induces HIF-1α expression by promoting transcription of two HIF-1α mRNA splice isoforms and driving increased protein translation in human and mouse T cells (37, 40). Additionally, PX-478 can prevent the G2/M transition by affecting proteins related to the G2 phase of the cell cycle, such as cyclin B1, thereby inhibiting cell proliferation (41).

Considering the inhibitory effects of PX-478 on T cell proliferation, we explored the potential of pre-treatment with PX-478 prior to CAR-T cell therapy. We pre-treated tumor cells with PX-478 and meticulously evaluated its influence on the proliferation and cytokine production of mesoCAR T cells. Proliferation analyses provided no significant differences in mesoCAR T cell proliferation between hypoxic and normoxic conditions, as well as PX-478-treated and untreated groups. Likewise, our analysis of IFN-γ and IL-2 cytokine production showed no significant differences, thereby confirming the results on proliferation. We further assessed how PX-478 affected the cytotoxicity of mesoCAR T cells. Consistent with previous research, no significant difference was observed in the cytotoxicity of mesoCAR T cells under hypoxic and normoxic conditions (11, 42). However, PX-478 significantly impeded the cytotoxic function of mesoCAR T cells. Earlier studies have indicated that HIF-1α is vital for the cytotoxic function of CAR T cells. For instance, Palazon et al. demonstrated that the deletion of HIF-1α resulted in reduced expression of several proteins critical for tumor rejection by cytotoxic T lymphocytes (43). The genetic ablation of HIF-1α led to decreased production of effector cytokines such as IFN-γ and TNF-α, along with cytolytic molecules like granzyme B (43). HIF-1α hydroxylation at proline residues in normoxia leads to VHL-mediated proteasomal degradation (44). It has been demonstrated that VHL-deficient TILs accumulate and survive in tumors in an HIF-dependent manner, retaining polyfunctionality and cytolytic capacity (45), highlighting the essential role of HIF-1α in T cells’ antitumor function.

HIFs have also been found to play a pivotal role in regulating T cell exhaustion in the context of infections and malignancies (43, 45–47). Consequently, we investigated the impact of PX-478 pre-treatment of tumor cells on the expression pattern of exhaustion markers on mesoCAR T cells. Our findings indicated an increase in the percentage of TIM3+ T cells and T_tex_ cells under hypoxia conditions and in PX-478 treated groups. This aligns with previous studies that have shown that the expression of TIM-3, a marker of terminally exhausted T cells, is substantially up-regulated under hypoxic conditions (43, 48, 49). The decrease in cytotoxicity of mesoCAR T cells in the presence of PX-478 may be explained by the increase in T_tex_ cells, which have limited antitumor activity.

Nevertheless, it is essential to acknowledge the limitations of our study. PX-478 may not be entirely specific for reducing HIF-1α levels; prior research suggests that it may affect other intracellular factors as well (50). In order to exclude off-target effects of PX-478, more specific approaches targeting HIF-1α, such as genetic knockdown or using alternative pharmacological inhibitors, would provide clearer evidence for the role of HIF-1α signaling in regulating antitumor function of mesoCAR T cells. Additionally, selectively rescuing HIF-1α protein levels in the presence of PX-478 using stabilizing agents that do not broadly impact other cell mediators would further elucidate the specific contribution of HIF-1α to the observed phenotypes. Additionally, HIF-1α is physiologically activated by hypoxia and plays a critical role in regulating the expression of several genes, including GLUT1, LDHA, and VEGF (12). Consequently, some downstream genes may exert either a positive or negative influence on the antitumoral function of CAR T cells. Further investigations may uncover specific downstream targets of HIF-1α that modulate the antitumor function of CAR T cells in the tumor microenvironment. Considering previous studies demonstrating HIF-1α can directly regulate expression of T cell activation-related genes such as CD69 in tumor-infiltrating lymphocytes (51), it would be informative to examine how PX-478 impacts levels of canonical activation markers on mesoCAR T cells.

PX-478 has previously demonstrated antitumor efficacy across several human tumor models (41, 52). However, our present investigation exclusively focuses on the *in vitro* evaluation of the potential combination therapy involving PX-478 and mesoCAR T cells. Our data demonstrate PX-478 partially impairs mesoCAR T cell function, but do not reflect the compound’s direct effects on cervical tumor cells or overall therapeutic potential *in vivo*. As we only assessed a subset of responses using an isolated cell system, our results should not be interpreted as evidence for negative impacts of HIF-1α inhibitors in cervical cancer more broadly. It is worth noting that our study was limited to *in vitro* assessments using cell lines and CAR T cell cocultures, while prior research has suggested that PX-478 may inhibit tumor angiogenesis, resulting in antitumor effects *in vivo.* Therefore, future *in vivo* studies are essential to provide a more comprehensive understanding of the function of PX-478 in a natural tumor microenvironment.

## 5 Conclusions

In summary, our study demonstrates the significant impact of HIF-1α inhibition using the PX-478 inhibitor on mesoCAR T cell function within the cervical cancer microenvironment. The inhibition of HIF-1α markedly impairs the cytotoxicity of mesoCAR T cells while minimally affecting their proliferation and cytokine production. Our findings underscore the clinical relevance of HIF-1α overexpression in cervical cancer patients and highlight the potential challenges in targeting HIF-1α for enhancing CAR T cell therapy efficacy. Despite limitations in specificity and the need for further *in vivo* validation, our study provides crucial insights into the interplay between HIF-1α signaling and CAR T cell function, serving as a foundational framework for the development of combination therapies aimed at optimizing CAR T cell performance in solid tumors like cervical cancer.

## Data availability statement

The original contributions presented in the study are included in the article/**Supplementary Material**. Further inquiries can be directed to the corresponding authors.

## Ethics statement

This study involves human cell lines and was approved by Research Ethics Committees of Biosafety & Laboratory, Tehran University of Medical Sciences [IR.TUMS.BLC.1402.015]. Informed consent was obtained from all individual participants included in the study.

## Author contributions

AP: Conceptualization, Data curation, Formal Analysis, Investigation, Visualization, Writing – original draft. BA: Conceptualization, Methodology, Project administration, Writing – original draft, Writing – review & editing. TS: Methodology, Writing – original draft. ZS: Investigation, Writing – original draft. MH: Investigation, Writing – original draft. JH: Supervision, Writing – review & editing. HM: Conceptualization, Funding acquisition, Supervision, Writing – review & editing.

## Supplementary material

Supplementary Figure 1 The effects of HIF-1α inhibitor PX-478 on the expression pattern of exhaustion markers. **(A)** The percentage of PD1+ T cells. **(B)** The frequency of T_pex_ and T_tex_ cells. **(C)** The percentage of TIM3+ T cells. Statistical analysis was performed using ordinary one-way ANOVA **(A-C)**, and Tukey multiple comparison test. *P < 0.05; **P < 0.01; ***P < 0.001. Data are presented as mean ± SD.
